# Does temporary ileostomy via specimen extraction site affect the short outcomes and complications after laparoscopic low anterior resection in rectal cancer patients? A propensity score matching analysis

**DOI:** 10.1186/s12893-022-01715-8

**Published:** 2022-07-07

**Authors:** Dong Peng, Dong-Ling Yu, Xiao-Yu Liu, Wei Tao, Bing Kang, Hua Zhang, Zheng-Qiang Wei, Guang-Yan Ji

**Affiliations:** 1 Department of Gastrointestinal Surgery, The First Affiliated Hospital of Chongqing Medical University, Chongqing, 400016 China; 2 Department of General Surgery, Qijiang District People’s Hospital, Chongqing, 401420 China; 3 Department of Clinical Nutrition, The First Affiliated Hospital of Chongqing Medical University, Chongqing, 400016 China

**Keywords:** Rectal cancer, Ileostomy, Laparoscopic low anterior resection, Propensity score matching, Specimen extraction

## Abstract

**Purpose:**

The purpose of the current study was to compare the outcomes of temporary stoma through the specimen extraction site (SSES) and stoma through a new site (SNS) after laparoscopic low anterior resection.

**Methods:**

The rectal cancer patients who underwent laparoscopic low anterior resection plus temporary ileostomy were recruited in a single clinical database from Jun 2013 to Jun 2020. The SSES group and the SNS group were compared using propensity score matching (PSM) analysis.

**Results:**

A total of 257 rectal cancer patients were included in this study, there were 162 patients in the SSES group and 95 patients in the SNS group. After 1:1 ratio PSM, there was no difference in baseline information (p > 0.05). The SSES group had smaller intraoperative blood loss (p = 0.016 < 0.05), shorter operation time (p < 0.01) and shorter post-operative hospital stay (p = 0.021 < 0.05) than the SNS group before PSM. However, the SSES group shorter operation time (p = 0.006 < 0.05) than the SNS group after PSM, moreover, there was no significant difference in stoma-related complications (p > 0.05). In the multivariate analysis, longer operation time was an independent factor (p = 0.019 < 0.05, OR = 1.006, 95% CI = 1.001–1.011) for the stoma-related complications.

**Conclusion:**

Based on the current evidence, the SSES group had smaller intraoperative blood loss, shorter operation time and shorter post-operative hospital stay before PSM, and shorter operation time after PSM. Therefore, SSES might be superior than SNS after laparoscopic low anterior resection for rectal cancer patients.

## Introduction

According to the World Health Organization, cancer is the leading cause of death globally, with approximately 18.1 million new cases diagnosed each year, which is expected to increase to 24 million by 2035 [1]. Colorectal cancer (CRC) is a major public health problem worldwide which ranks the third most common cancers, with nearly 1.9 million new cases of CRC detected each year, and CRC is responsible for 916,000 deaths every year and is the second leading cause of cancer-related death [2–5].

CRC can be divided into rectal cancer and colon cancer. For patients with lower rectal cancer, temporary ileostomy is often performed to minimize the risk of anastomotic complications including leakage and re-operation after rectal resection [6–8]. Patients with temporary ileostomy have a lower risk of developing anastomotic leakage and peritonitis than patients without temporary ileostomy [9–12].

There were two methods in terms of the site of temporary ileostomy: temporary ileostomy through the specimen extraction site (SSES) and stoma through a new site (SNS) after laparoscopic low anterior resection. However, it remained controversial which site of temporary ileostomy was better [13–17]. Some studies reported there was no difference between SSES and SNS [17], however, other studies reported SSES was a better method [13–15]. Therefore, the purpose of the current study aims to compare the outcomes of temporary ileostomy through SSES and SNS after laparoscopic low anterior resection.

## Methods

### Patients

The rectal cancer patients who underwent laparoscopic low anterior resection plus temporary ileostomy were recruited in a single clinical database from Jun 2013 to Jun 2020. The study was approved by the ethics committee of local institution (The First Affiliated Hospital of Chongqing Medical University, 2021-519), and all patients signed informed consent forms. This study was conducted in accordance with the World Medical Association Declaration of Helsinki as well.

### Inclusion and exclusion criteria

**Fig. 1:**
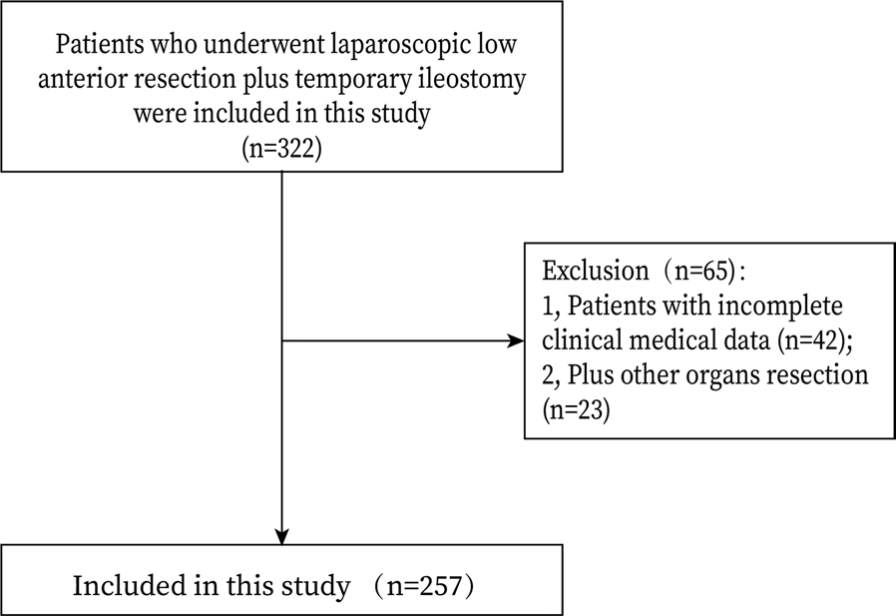
Flow chart of patient selection

Patients who were diagnosed with CRC and underwent laparoscopic low anterior resection plus temporary ileostomy were included in this study (n = 322). The exclusion criteria were as follows: 1, Patients with incomplete clinical medical data (n = 42); and 2, Plus other organs resection (n = 23). Finally, a total of 257 patients were included in this study. (Fig. 1).

### Surgery management and Definitions

**Fig. 2:**
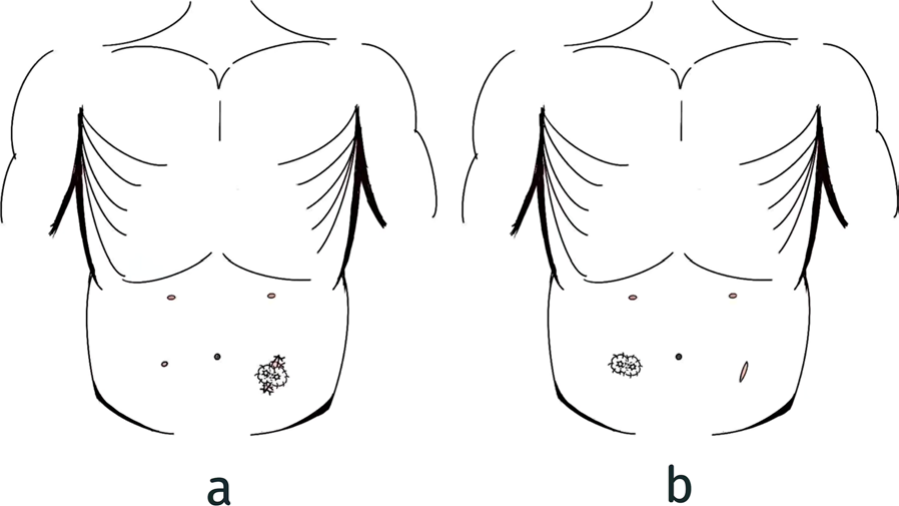
The site of temporary ileostomy. **a** SSES; **b** SNS. *SSES* stoma through the specimen extraction site, *SNS* stoma through a new site

The tumor stage was diagnosed according to the AJCC 8^th^ Edition [18]. The laparoscopic low anterior resection plus in temporary ileostomy was according to the principles of oncology, the positions of the five trocars were as follows: one trocar was punctured up the umbilicus, two trocars were punctured at left side of abdomen and the other two trocars were punctured at right side of abdomen. Placing a wound protector before specimen removal. Find the end of the ileum under laparoscopy (40 cm from the left temporary ileostomy and 20 cm from the right temporary ileostomy), and use absorbable sutures to suture the bowel with the peritoneum, the anterior sheath and the skin layer intermittently, then temporary ileostomy was performed. The temporary ileostomy was divided into two groups: SSES and SNS. The SSES group was defined as the temporary ileostomy was located at the specimen extraction site and the SNS group was defined as the temporary ileostomy was located at a new site. (Fig. 2) Postoperative complications were graded by the Clavien-Dindo classification [19], and the major complications were defined as ≥ grade III, which required surgery, endoscopy or radiological intervention.

### Data collection

The clinical characteristics were collected in the inpatient system, outpatient system and telephone interviews. The baseline information included age, sex, body mass index (BMI), smoking, drinking, hypertension, type 2 diabetes mellitus (T2DM), coronary heart disease (CHD), pre-operative hemoglobin, pre-operative albumin, neoadjuvant chemoradiation, stoma formation (SSES and SNS) and tumor nodes metastasis (TNM) stage. The outcomes included intraoperative blood loss, operation time, post-operative hospital stay, post-operative overall complications, post-operative major complications and stoma-related complications (The time from laparoscopic low anterior resection plus in temporary ileostomy to stoma retraction).

### PSM

To minimize the bias of baseline characteristics of the SSES group and the SNS group, PSM was conducted. Nearest neighbor matching was performed without replacement at a 1:1 ratio and a caliper width with a 0.2 standard deviation was specified. The matched baseline information was as follows: age, sex, BMI, drinking, smoking, T2DM, hypertension, CHD, pre-operative hemoglobin, pre-operative albumin, neoadjuvant chemoradiation and TNM stage.

### Statistical analysis

Continuous variables are expressed as the mean ± SD and independent-sample t test was used. Frequency variables are expressed as n (%), and Chi-square test or Fisher's exact test was used. The univariate logistic regression was conducted to find potential predictors for stoma-related complications, the p value < 0.1 and clinical important risk factors were included in the final multivariate logistic regression for independent risk factors. Data were analyzed using SPSS (version 22.0) statistical software. A bilateral p value of < 0.05 was considered statistically significant.

## Results

### Patients

A total of 257 rectal cancer patients were included in this study, the rectal cancer patients who underwent laparoscopic low anterior resection plus temporary ileostomy and no conversion occurred. The average age was 61.3 ± 10.8 years old. There were 163 (63.4%) males and 94 (36.6%) females. The other clinical characteristics were summarized in Table 1.

**Table 1:** Clinical characteristics of rectal cancer patients

Characteristics	No. 257
Age (mean ± SD), year	61.3 ± 10.8
Sex	
Male	163 (63.4%)
Female	94 (36.6%)
BMI (mean ± SD), kg/m^2^	22.9 ± 3.0
Smoking	110 (42.8%)
Drinking	88 (32.4%)
Hypertension	64 (24.9%)
T2DM	25 (9.7%)
CHD	7 (2.7%)
Pre-operative hemoglobin, g/L	126.7 ± 19.7
Pre-operative albumin, g/L	40.8 ± 4.8
Neoadjuvant chemoradiation	73 (28.4%)
Stoma formation	
SSES	162 (63.0%)
SNS	95 (37.0%)
TNM stage	
I	94 (36.6%)
II	74 (28.8%)
III	81 (31.5%)
IV	8 (3.1%)

Variables are expressed as the mean ± SD, n (%), *P-value < 0.05

*T2DM* type 2 diabetes mellitus, *BMI* body mass index, *CHD* coronary heart disease, *SSES* stoma through the specimen extraction site, *SNS* stoma through a new site; *TNM* tumor nodes metastasis

### Baseline characteristics before and after PSM

There were 162 patients in the SSES group and 95 patients in the SNS group. Baseline information including age, sex, BMI, smoking, drinking, hypertension, T2DM, CHD, pre-operative hemoglobin, pre-operative albumin and TNM stage were compared before and after 1:1 ratio PSM. The pre-operative albumin was 41.6 ± 4.7 g/L in the SSES group which was significantly higher than 40.3 ± 4.9 g/L in the SNS group (p = 0.041 < 0.05) before PSM. Therefore, PSM was conducted and there was no significant difference between the two groups (p > 0.05) in baseline information after PSM. (Table 2).

**Table 2:** Baseline characteristics before and after PSM

Characteristics	Before PSM			After PSM		
	SSES (162)	SNS (95)	P value	SSES (95)	SNS (95)	P value
Age, year	62.0 ± 10.3	60.3 ± 11.5	0.225	61.8 ± 10.2	60.3 ± 11.5	0.322
Sex			0.639			0.759
Male	101 (62.3%)	62 (65.3%)		64 (67.4%)	62 (65.3%)	
Female	61 (37.7%)	33 (34.7%)		31 (32.6%)	33 (34.7%)	
BMI, kg/m^2^	22.9 ± 2.9	22.9 ± 3.1	0.915	23.1 ± 2.8	22.9 ± 3.1	0.708
Smoking	70 (43.2%)	40 (42.1%)	0.863	44 (46.3%)	40 (42.1%)	0.559
Drinking	55 (34.0%)	33 (34.7%)	0.898	37 (38.9%)	33 (34.7%)	0.547
Hypertension	39 (24.1%)	25 (26.3%)	0.688	23 (24.2%)	25 (26.3%)	0.738
T2DM	15 (9.3%)	10 (10.5%)	0.741	9 (9.5%)	10 (10.5%)	0.809
CHD	6 (3.7%)	1 (1.1%)	0.265	1 (1.1%)	1 (1.1%)	1.000
Pre-operative hemoglobin, g/L	125.7 ± 19.4	128.4 ± 20.4	0.302	128.0 ± 19.6	128.4 ± 20.4	0.908
Pre-operative albumin, g/L	40.3 ± 4.9	41.6 ± 4.7	0.041*	40.5 ± 4.8	41.6 ± 4.7	0.099
Neoadjuvant chemoradiation	48 (29.6%)	25 (26.3%)	0.668	28 (29.5%)	25 (26.3%)	0.627
TNM stage			0.100			0.053
I	55 (34.0%)	39 (41.0%)		33 (34.7%)	39 (41.0%)	
II	54 (33.3%)	20 (21.1%)		35 (36.8%)	20 (21.1%)	
III	50 (30.9%)	31 (32.6%)		26 (27.4%)	31 (32.6%)	
IV	3 (1.8%)	5 (5.3%)		1 (1.1%)	5 (5.3%)	

*T2DM* type 2 diabetes mellitus, *CHD* coronary heart disease, *BMI* body mass index, *PSM* propensity score matching, *SSES* stoma through the specimen extraction site, *SNS* stoma through a new site, *TNM* tumor nodes metastasis

Variables are expressed as the mean ± SD, n (%), *P-value < 0.05

### Outcomes

The outcomes including intraoperative blood loss, operation time, post-operative hospital stay, post-operative overall complications, post-operative major complications and stoma-related complications (stoma edema, stoma prolapse, stoma necrosis, stoma bleeding, stoma stenosis, parastomal hernia and skin inflammation around the stoma) were compared before and after PSM. Although stoma complications occurred, no patients underwent reestablishment of stoma.

Before PSM, the intraoperative blood loss was 71.6 ± 67.9 mL in the SSES group which was smaller than 100.0 ± 119.2 mL in the SNS group (p = 0.016 < 0.05). The operation time was 235.5 ± 76.2 min in the SSES which was shorter than 274.7 ± 77.0 min in the SNS group (p < 0.01). The post-operative hospital stay was 8.0 ± 4.0 days in the SSES which was shorter than 9.5 ± 6.0 days in the SNS group (p = 0.021 < 0.05). There was no significant difference in stoma-related complications (p > 0.05).

After PSM, the SSES group had shorter operation time (p = 0.006 < 0.05) than the SNS group. There was no significant difference in stoma-related complications (p > 0.05). (Table 3).

**Table 3:** Outcomes before and after PSM

Characteristics	Before PSM			After PSM		
	SSES (162)	SNS (95)	P value	SSES (95)	SNS (95)	P value
Intraoperative blood loss, mL	71.6 ± 67.9	100.0 ± 119.2	0.016*	76.0 ± 63.5	100.0 ± 119.2	0.085
Operation time, min	235.5 ± 76.2	274.7 ± 77.0	< 0.01**	243.1 ± 78.5	274.7 ± 77.0	0.006**
Post-operative hospital stay, day	8.0 ± 4.0	9.5 ± 6.0	0.021*	8.4 ± 4.7	9.5 ± 6.0	0.182
Post-operative overall complications	34 (21.0%)	26 (27.4%)	0.243	22 (23.2%)	26 (27.4%)	0.504
Post-operative major complications	1 (0.6%)	4 (4.2%)	0.064	0 (0.0%)	4 (4.2%)	0.121
Stoma-related complications	25 (0.6%)	11 (11.6%)	0.390	17 (17.9%)	11 (11.6%)	0.219
Stoma edema	0 (0.0%)	2 (2.1%)	0.136	0 (0.0%)	2 (2.1%)	0.497
Stoma prolapse	1 (0.6%)	0 (0.0%)	1.000	0 (0.0%)	0 (0.0%)	–
Stoma necrosis	0 (0.0%)	1 (1.1%)	0.370	0 (0.0%)	1 (1.1%)	1.000
Stoma bleeding	2 (1.2%)	0 (0.0%)	0.532	1 (1.1%)	0 (0.0%)	1.000
Stoma stenosis	2 (1.2%)	0 (0.0%)	0.532	2 (2.1%)	0 (0.0%)	0.497
Skin inflammation around the stoma	16 (9.9%)	6 (6.3%)	0.325	11 (11.6%)	6 (6.3%)	0.204
Parastomal hernia	4 (2.5%)	2 (2.1%)	1.000	3 (3.2%)	2 (2.1%)	1.000

*PSM* propensity score matching, *SSES* stoma through the specimen extraction site, *SNS* stoma through a new site

Variables are expressed as the mean ± SD, n (%), *P-value < 0.05, **P-value < 0.01

### Univariate and multivariate analysis of the stoma-related complications

Univariate analysis was conducted to find potential factors for the stoma-related complications, and we found that longer operation time was a potential factor (p = 0.038 < 0.05, OR = 1.005, 95% CI = 1.000–1.010) for the stoma-related complications. Furthermore, in the multivariate analysis, longer operation time was an independent factor (p = 0.019 < 0.05, OR = 1.006, 95% CI = 1.001–1.011). (Table 4).

**Table 4:** Univariate and multivariate analysis of the stoma-related complications

Risk factors	Univariate analysis		Multivariate analysis	
	OR (95% CI)	P value	OR (95% CI)	P value
Age, year	1.008 (0.971–1.046)	0.673		
Sex (male/female)	0.757 (0.314–1.828)	0.536		
BMI, Kg/m^2^	1.013 (0.884–1.160)	0.858		
Hypertension (yes/no)	1.220 (0.499–2.984)	0.663		
T2DM (yes/no)	1.095 (0.297–4.034)	0.892		
TNM stage (IV/III/II/I)	0.921 (0.587–1.447)	0.722		
Smoking (yes/no)	1.314 (0.589–2.935)	0.505		
Drinking (yes/no)	1.130 (0.496–2.573)	0.772		
CHD (yes/no)	5.963 (0.362–98.217)	0.212		
Pre-operative hemoglobin, g/L	1.002 (0.982–1.022)	0.866		
Pre-operative albumin, g/L	0.996 (0.915–1.084)	0.925		
Operation time, min	1.005 (1.000–1.010)	0.038*	1.006 (1.001–1.011)	0.019*
Intraoperative blood loss, mL	1.001 (0.997–1.005)	0.565		
Neoadjuvant chemoradiation	1.040 (0.427–2.530)	0.931		
Stoma formation (SSES/ SNS)	1.664 (0.734–3.774)	0.223	2.023 (0.864–4.736)	0.105

*OR* Odds ratio, *CI* confidence interval, *BMI* body mass index, *T2DM* type 2 diabetes mellitus, *CHD* coronary heart disease, *SSES* stoma through the specimen extraction site, *SNS* stoma through a new site, *TNM* tumor nodes metastasis

*P-value < 0.05, **P-value < 0.01

## Discussion

A total of 257 rectal cancer patients were included in this study, there were 162 patients in the SSES group and 95 patients in the SNS group. After 1:1 ratio PSM, there was no difference in baseline information. The SSES group had smaller intraoperative blood loss, shorter operation time and shorter post-operative hospital stay than the SNS group before PSM, and shorter operation time after PSM. However, there was no significant difference in stoma-related complications. In the multivariate analysis, longer operation time was an independent factor for stoma-related complications.

Anastomotic leakage remains a major problem after laparoscopic anterior resection, with an incidence of 1.4%–15.2% [20–23]. In some cases, anastomotic leakage could lead to devastating consequences including peritonitis, pelvic abscess, and rectovaginal fistula [24, 25]. Prophylactic stoma was often required after laparoscopic low rectal cancer surgery [7]. During the laparoscopic low rectal cancer surgery, a small incision was often required to remove the specimen and prophylactic stoma was made through the specimen extraction site or through a new site. Prophylactic stoma could reduce the occurrence of anastomotic leakage and reoperation [6–8].

We summarize the detailed viewpoints in Table 5 concerning the difference between SSES and SNS. Some studies reported that there was no statistically significant difference between the SSES group and SNS group in all stoma related complications [13, 14, 17]. However, Li W et al. [15] reported the SSES had group had a lower parastomal hernia rate. Karakayali FY et al. [16] reported the SNS group had lower parastomal hernia rate than the SSES group. As for other surgical outcomes including operation time, post-operative hospital stay, it remained controversial as well [13–17]. Therefore, it is important to analyze the surgical outcomes and stoma related complications elaborately. Furthermore, PSM was conducted to reduce the selection bias, which could benefit precise results when there was no difference in baseline information [26, 27].

**Table 5:** Previous studies reporting the difference between the SSES group and the SNS group

Author	Year	Country	Sample size	SSES	SNS	Outcomes
Lee KY et al. [12]	2019	Korea	198	141	57	The SSES group had a shorter operation time and was associated with fewer cases of wound infection than the SNS group. There was no statistically significant difference between the SSES group and SNS group in all-stoma complications
Wang P et al. [13]	2018	China	331	155	176	The SSES group had a shorter operation time, less estimated blood and wound infections than the SNS group. The estimated 5-year disease-free survival and overall survival rate were similar between the two groups. There was no statistically significant difference between the SSES group and SNS group in all-stoma complications
Li W et al. [14]	2017	China	738	139	599	The SSES had lower parastomal hernia rate, a shorter operation time, less estimated blood and all-stoma complications than the SNS group
Karakayali FY et al. [15]	2015	Turkey	46	21	25	The SNS group had shorter hospital stay, shorter time to resumption of regular diet and lower parastomal hernia rate than the SSES group
Yoo SB et al. [16]	2013	Korea	105	56	49	No significant difference was found between the SSES group and SNS group in terms of all-stoma complications

*SSES* stoma through the specimen extraction site, *SNS* stoma through a new site

In this study, we found that the SSES group had smaller intraoperative blood loss, shorter operation time and shorter post-operative hospital stay than the SNS group before PSM, and the SSES group had shorter operation time after PSM. These results were similar with previous studies [13–16], the possible reason was that the SSES group omitted the step of suturing the incision, which greatly reduced the operation time.

As for stoma-related complications, there was no significant difference between the SSES group and SNS group. Previous studies had controversial outcomes of parastomal hernia between the two groups [15, 16]. Our study indicated that the SSES group and SNS group had similar stoma-related complications.

Moreover, In this study, multivariate logistic regression was used for analyzing independent risk factors of stoma-related complications and we found that longer operation time was an independent predictor of stoma-related complications. The reason was unclear, but it might be related to the difficulty of surgery and the difficulty of stoma formation. We hypothesized that the stoma-related complications were mainly based on the the difficulty of stoma formation, however the baseline characteristics or stoma formation did not affect the outcomes. Therefore, cautious and skilled operative procedures were necessary for surgeons.

To our knowledge, this is the first study analyzing the difference between the SSES group and the SNS group using PSM. Furthermore, we conducted the multivariate logistic analysis to find independent predictive factors of stoma-related complications for the first time.

Our study had some limitations. First, this was a single retrospective study which might cause selection bias (SSES and SNS might not be randomly selected), therefore, we conducted PSM to minimize the difference of baseline information; Second, long-term survival outcomes were lacking; Third, the sample size in this study was relatively small, some parameters such as renal function and blood electrolytes after stoma formation were not analyzed; Fourth, the operation time of stoma formation was missing as well, and the site of the temporary ileostomy was not marked before surgery which might result in non-standardized stoma formation. Therefore, larger sample size with detailed information and long-term follow-up should be conducted in the following experiments.

In conclusion, based on the current evidence, the SSES group had smaller intraoperative blood loss, shorter operation time and shorter post-operative hospital stay before PSM, and shorter operation time after PSM. Therefore, SSES might be superior than SNS after laparoscopic low anterior resection for rectal cancer patients.

## Data Availability

The datasets generated and/or analysed during the current study are not publicly available due [The database from our clinical center were relatively private] but are available from the corresponding author on reasonable request.
